# MSST-RT: Multi-Stream Spatial-Temporal Relative Transformer for Skeleton-Based Action Recognition

**DOI:** 10.3390/s21165339

**Published:** 2021-08-07

**Authors:** Yan Sun, Yixin Shen, Liyan Ma

**Affiliations:** 1 School of Computer Engineering and Science, Shanghai University, 99 Shangda Road, Shanghai 200444, China; shenyixin@shu.edu.cn (Y.S.); liyanma@shu.edu.cn (L.M.); 2 Shanghai Institute for Advanced Communication and Data Science, 333 Nanchen Road, Shanghai 200444, China

**Keywords:** action recognition, 3D skeleton, transformer, attention, spatial-temporal

## Abstract

Skeleton-based human action recognition has made great progress, especially with the development of a graph convolution network (GCN). The most important work is ST-GCN, which automatically learns both spatial and temporal patterns from skeleton sequences. However, this method still has some imperfections: only short-range correlations are appreciated, due to the limited receptive field of graph convolution. However, long-range dependence is essential for recognizing human action. In this work, we propose the use of a spatial-temporal relative transformer (ST-RT) to overcome these defects. Through introducing relay nodes, ST-RT avoids the transformer architecture, breaking the inherent skeleton topology in spatial and the order of skeleton sequence in temporal dimensions. Furthermore, we mine the dynamic information contained in motion at different scales. Finally, four ST-RTs, which extract spatial-temporal features from four kinds of skeleton sequence, are fused to form the final model, multi-stream spatial-temporal relative transformer (MSST-RT), to enhance performance. Extensive experiments evaluate the proposed methods on three benchmarks for skeleton-based action recognition: NTU RGB+D, NTU RGB+D 120 and UAV-Human. The results demonstrate that MSST-RT is on par with SOTA in terms of performance.

## 1. Introduction

Human action recognition attracts extensive attention due to its important application prospects in many fields, such as healthcare assistance, human–computer interaction and autonomous driving. Many exciting developments have taken place in the study of this research topic in recent years. A consensus has been reached that the key to human action recognition is learning how to capture sufficient spatial-temporal information.

Early research in this field was mostly based on RGB videos. However, RGB videos are often affected by the variations in illuminations, changes in camera viewpoints and background noise. All of these are obstacles in the process of extracting discriminative features. Compared with RGB data, the skeleton is free from these difficulties; it also has fewer restrictions in terms of data size. For example, it provides a compact form to represent dynamic information. These advantages make it possible to design lightweight models based on skeleton data. Meanwhile, with the development of human motion estimation technologies, such as advanced human pose estimation algorithms [1] and multimodal sensors [2], skeleton data are easier and cheaper to obtain than before, which inspires researchers to explore various approaches based on skeleton data for action recognition. In this paper, the action recognition research is based on 3D skeleton data.

To extract information from skeleton data, researchers have mainly explored four aspects: handcrafted-features-based methods, convolution neural-network-based methods, recurrent neural-network-based methods and graph convolution-network-based methods. In recent years, the graph convolution network (GCN) has become the most widespread method, with the characteristics of low computation and high accuracy. Although these methods have achieved great success in terms of performance, there some defects still exist: (1) Most human actions are performed by a collaborative effort among the joints that not linked in the human skeleton. For example, the left hand and right hand are not linked, but they have a strong correlation in the action of “clapping”. Graph convolutions only gather information from the local neighbor joints of two hands, respectively, but neglect the relationship between hands. Applying graph convolution repeatedly would obtain a multi-hop dependence between the two hands; however, this would occur at the expense of increasing computational complexity and would make optimization more difficult. (2) It is hard to achieve long-range dependence utilizing only graph convolution in the model, although it plays an important role in temporal dimension. (3) The rich information derived from different scales of motion can effectively supplement each other, but this is usually neglected in the research.

In this paper, we propose a novel mechanism with a lightweight transformer to overcome the first and second limitations mentioned in the previous paragraph, and call it a relative transformer. The relative transformer mechanism is employed in spatial skeleton-based architecture to build bridges between two distant joints and propagate signals. It is also employed in the temporal dimension to capture long-range interactions between two distant frames. As a consequence, the model is named the spatial-temporal relative transformer (ST-RT). For the third defect, we also propose an effective dynamic representation which fuses three different scales of motion and raw position to obtain richer information from a skeleton sequence in ST-RT.

**Figure 1:**
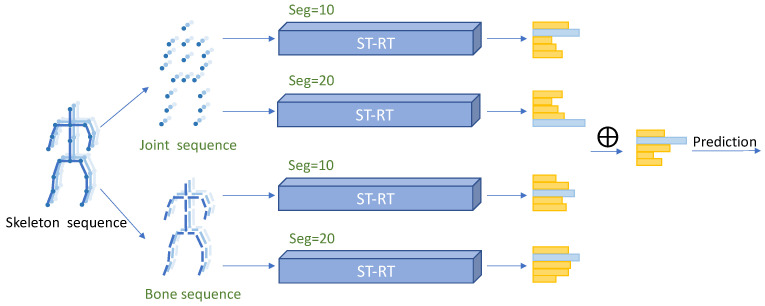
Illustration of the overall architecture of the proposed MSST-RT. The sum of all scores from four ST-RTs is treated as the final prediction.

The overall architecture of the proposed MSST-RT is shown in Figure 1. As well as the joint information, the bone information derived from the joints’ positions is also learned by ST-RT. Furthermore, the skeleton sequence, obtained by different sampling strategies, provides supplementary information through model training, e.g., 10 and 20 frames are sampled from the joint sequence and bone sequence. Four ST-RTs are combined to extract features from two joint sequences and two bone sequences. We name this the multi-stream spatial-temporal relative transformer (MSST-RT). Significantly, batch normalization is adopted instead of layer normalization in relative transformer to obtain a faster training time and higher performance.

There are five main contributions of this work, summarized as follows:

- We propose an MSST-RT model comprising four ST-RTs. Each of them extracts features from a corresponding skeleton sequence, which complement each other. It is worth noting that MSST-RT eschews recurrence and graph convolution and instead relies entirely on a relative transformer mechanism to learn long-distance dependencies.
- A multi-scale dynamic representation (DR) combines various scale-of-motion features from one skeleton sequence.
- A lightweight relative transformer module is designed for spatial and temporal modeling. Regarding the spatial dimension, a spatial relative transformer module (SRT) is introduced to establish long-range dependencies while maintaining the origin skeleton topology. In the temporal dimension, the temporal relative transformer module (TRT) studies long-range interaction between nonadjacent frames, with the order of skeleton sequence remaining unchanged.
- Attention responses in SRT and TRT are visualized to show the effects of the attention mechanism. This proves that the proposed ST-RT pays great attention to some nodes and the distribution of attention is consistent with human perception.
- Our MSST-RT achieves state-of-the-art on three benchmark datasets, i.e., NTU RGB+D, NTU RGB+D 120 and UAV-Human, in action recognition based on skeleton.

## 2. Related Work

### 2.1. Skeleton-Based Action Recognition

Action recognition based on skeleton can be summarized into two major categories, i.e., traditional methods [3,4,5] that use hand-crafted features and deep learning methods. In recent years, deep neural networks have gained increasing favor due to their remarkable performance in the following: (1) RNN-based methods [6,7,8,9,10] model the contextual information by splicing the coordinates of the key points at each moment into a vector and concatenating all vectors for each frame. For example, H. Wang and W. Liang introduce a two-stream RNN [7] architecture which incorporates both spatial and temporal RNN networks for skeleton based action recognition. The HBRNN-L model [9] decompose the human skeleton into five parts, and then feed them to five bidirectional RNN subnets. (2) CNN-based methods [11,12,13,14,15] usually reconstructed the skeleton sequenceto a series of pseudo-images to obtain the spatial cues. Wang et al. [16] propose a novel CNN network, which represents the spatial configuration and dynamic information of a joint trajectory as three texture images by color encoding. (3) GCN-based methods [17,18,19] preserve the inherent topological graph data struct of the skeleton by treating joints and bones as vertices and edges. Taking advantage of the graph data struct, GCN-based methods build operators in the non-Euclidean space and outperform the other two approaches. In particular, the ST-GCN model proposed by Yan et al. [17] is the first construct, a spatial-temporal graph that offers a new partitioning strategy.A lot of other GCN-based methods regard it as a baseline, or improve on it.

### 2.2. Transformer

Transformer [20] is a novel architecture, which has now become the standard for NLP tasks. It handles long-range dependencies by relying on self-attention rather than sequence-aligned RNNs or convolution. Recently, many transformer-based models have been proposed to improve the original architecture, e.g., Set Transformer [21], Routing Transformer [22] and Star-Transformer [23]. Most of them aim to overcome the computation complexity and large memory overhead. In addition to being developed for NLP, transformers became a research hotspot in the fields of Computer Vision. Carion et al. proposed a Detection Transformer (DETR) [24], which is the first object detection framework that combines the convolutional neural network with a transformer. Vision Transformer (ViT) [25] utilizes transformer architecture without CNN and it outperforms the state-of-the-art convolutional network in various image classification tasks. In our work, the model only consists of relative transformer modules. Inspired by the Star-Transformer, the proposed relative transformer is a variation based on standard transformer architecture designed for skeleton action recognition.

## 3. Background

The transformer model mainly includes an encoding component and decoding component. Our work is concerned with the encoding part, which is broken down into a self-attention layer and feed-forward neural network layer. This section will briefly review the relevant knowledge.

### 3.1. Attention in the Encoding

In NLP tasks, each word has a query vector q, a key vector k and a value vector v to calculate a score, which represents the effect of other words on the input sentence on the encoding of the word. The score is calculated by taking the dot product $s_{ij}=q_{i}\cdot k_{j}^{T}\;i,j=1,\cdots ,n$. The output of the self-attention layer is obtained by multiplying each value vector by the score and summing up the weighted value vectors. While packing all query vectors into a matrix *Q*, all key vectors into a matrix *K* and all value vectors into a matrix *V*, the attention function is defined as:

(1) $$Attention\left(Q,K,V\right)=softmax\left(\frac{QK^{T}}{\sqrt{d_{k}}}\right)V$$

where $\frac{1}{\sqrt{d_{k}}}$ leads to a more stable gradient. Furthermore, the “multi-headed” attention initializes multiple sets of weight matrices randomly and obtains different scores. Concatenating the scores as the result helps the network capture richer features.

### 3.2. Feed-Forward Neural Network in the Encoding

In addition to the attention layer, the encoder also contains a feed-forward neural network, which consists of two linear transformations and an ReLU activation:

(2) $$FFN\left(x\right)=max\left(0,xW_{1}+b_{1}\right)W_{2}+b_{2}$$

The network projects the refined vector, which was obtained from multi-head attention, into a larger space to improve the ability to capture information. We also apply it in our relative transformer network.

## 4. Multi-Stream Spatial-Temporal Relative Transformer Network (MSST-RT)

In order to solve the limitations of the small receptive field in traditional convolution, a Transformer is introduced to the skeleton-based action recognition models instead of graph convolution. Since the graph is in an unordered sequence, we propose a new transformer architecture, named the relative transformer to keep the topology of the graph with lower complexity. The proposed multi-stream spatial-temporal relative transformer network (MSST-RT) consists of four spatial-temporal relative transformer networks (ST-RT). Four kinds of skeleton sequence, including a joint sequence of 10 sampled frames, a joint sequence of 20 sampled frames, a bone sequence of 10 sampled frames and a bone sequence of 20 sampled frames (shown in Figure 1). They are fed into four ST-RTs for feature extraction, fusion and prediction.

**Figure 2:**
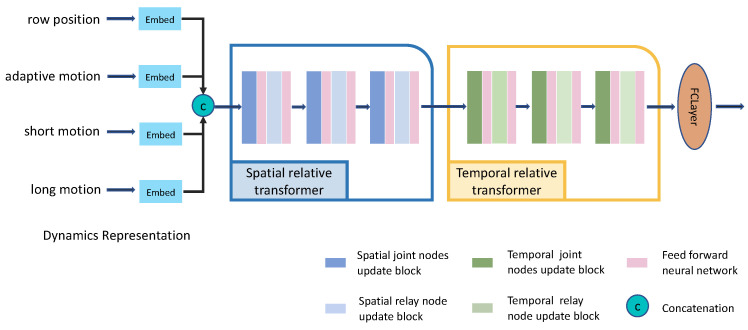
Illustration of the spatial-temporal relative transformer (SR-RT). The skeleton data are processed by three modules and then fed into the fully connected layer to predict the score for each action class.

In this section, we will introduce our model ST-RT, where relative transformers are employed in both space and temporal dimensions; the model architecture is illustrated in Figure 2. It consists of three modules: dynamics representation (DR), spatial relative transformer (SRT) and temporal relative transformer (TRT). Meanwhile, each relative transformer module contains three node update blocks and each block is subdivided into two sub-blocks: joint nodes update block and relay node update block. The feed-forward neural network (FFN) is connected behind them in both a spatial and temporal relative transformer.

### 4.1. Dynamics Representation (DR)

**Figure 3:**
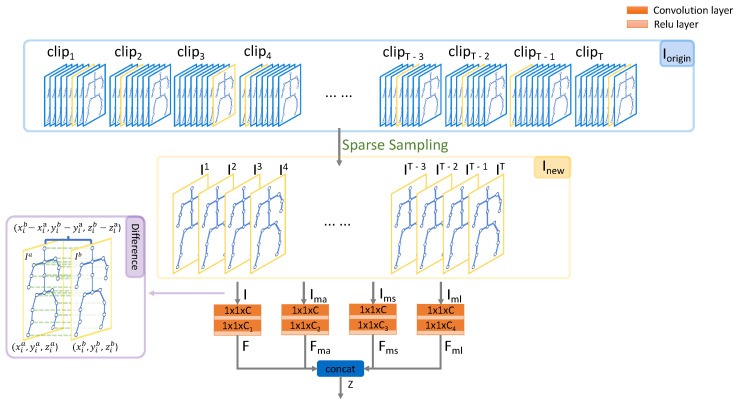
Illustration of dynamics representation (DR). There are four streams of skeleton information embedded into a higher dimension by the embedding block and then concatenated as an input of the spatial relative transformer. Each block consists of two convolution layers and two activation layers.

Temporal difference operations are always adopted for motion extraction in action recognition, such as TEINet [26] and STM [27]. In action recognition, all the 3D positions of skeleton joints in NTU60 and NTU120 datasets are stored, and UAV-Human stores 2D positions. Figure 3 shows the 3D positions of skeleton joints. A joint in frame *a* is represented as $J_{i}^{a}=\left(x_{i}^{a},y_{i}^{a},z_{i}^{a}\right)$, the same joint in frame b(b>a) is $J_{i}^{b}=\left(x_{i}^{b},y_{i}^{b},z_{i}^{b}\right)$. The temporal difference is the subtraction between the same joint in the two frames, which can be denoted as $\left(x_{i}^{b}-x_{i}^{a},y_{i}^{b}-y_{i}^{a},z_{i}^{b}-z_{i}^{a}\right)$.In view of the significant motion variations in actions, we combine different scale motions to model the temporal information of action, as shown in Figure 3. This operation improves the generalization of our network, as the fixed motion focuses on different ranges of motion while the adaptive motion focuses on different durations.

In more detail, we divide the original sequence $I_{origin}=\left[I^{1},\cdots ,I^{F}\right]$ into *T* equal clips and randomly sample one frame from each clip to form a new sequence $I_{new}=\left[I^{1},\cdots ,I^{T}\right]$ in order. The original sequence $I_{origin}$ is either a joint sequence or bone sequence. The bone extractor proposed by Shi et al. [28] is applied to datasets to obtain bone information, which ensures that the representation of both the bone sequence and joint sequence are exactly the same. *I* represents the row positions of all joint ponits in single frame. The motion is computed by taking the difference of each joint node $Jo_{i}^{t}\left(Jn_{i}^{t}\right)$ between two frames: $Jo_{i}^{t}$ denotes the *i*-th joint node in frame *t* of $I_{origin}$ and $Jn_{i}^{t}$ denotes the *i*-th joint node in frame *t* of $I_{new}$. The adaptive motion $I_{ma}$ is the difference between consecutive frames in $I_{new}$, which represents different scales of motion information in the unequal video:

(3) $$I_{ma}=\left\{I_{ma}^{1},I_{ma}^{2},\cdots ,I_{ma}^{T}\right\}$$

(4) $$I_{ma}^{t}=\left\{Jn_{1}^{t+1}-Jn_{1}^{t},Jn_{2}^{t+1}-Jn_{2}^{t},\cdots ,Jn_{N}^{t+1}-Jn_{N}^{t}\right\}\;t=1,2,\cdots ,T$$

where $I_{ma}^{t}$ denotes the adaptive motion of frame *t* in the new sequence. Note that although the difference is between the adjacent frames in $I_{new}$, the distance between these two frames depends on their location of $I_{origin}$, which is interconnected with the length of the skeleton sequence. Hence, each skeleton sequence obtains an adaptive scale motion based on length.

Furthermore, there are two types of fixed motion: short-scale $I_{ms}$ and long-scale $I_{ml}$. The function is expressed in the following:

(5) $$I_{ms}=\left\{I_{ms}^{1},I_{ms}^{2},\cdots ,I_{ms}^{T}\right\}$$

(6) $$I_{ml}=\left\{I_{ml}^{1},I_{ml}^{2},\cdots ,I_{ml}^{T}\right\}$$

(7) $$I_{ms}^{t}=\left\{Jo_{1}^{f+3}-Jo_{1}^{f},Jo_{2}^{f+3}-Jo_{2}^{f},\cdots ,Jo_{N}^{f+3}-Jo_{N}^{f}\right\}\;t=1,2,\cdots ,T$$

(8) $$I_{ml}^{t}=\left\{Jo_{1}^{f+6}-Jo_{1}^{f},Jo_{2}^{f+6}-Jo_{2}^{f},\cdots ,Jo_{N}^{f+6}-Jo_{N}^{f}\right\}\;t=1,2,\cdots ,T$$

where $I_{ms}^{t}$ denotes the short motion of frame *t* in the original sequence and $I_{ml}^{t}$ is the long motion of frame *t* in the original sequence. *f* represents the frame number in the original video.

Finally, the row position and three different types of motion are embedded into the high-dimension tensor, i.e., $F^{t}$, $F_{ma}^{t}$, $F_{ms}^{t}$ and $F_{ml}^{t}$, and concatenate them.

(9) $$Z^{t}=concat\left(\left[F^{t},F_{ma}^{t},F_{ms}^{t},F_{ml}^{t}\right]\right)=\left\{Z_{1}^{t},Z_{2}^{t},\cdots ,Z_{N}^{t}\right\}$$

(10) $$Z=\{Z^{1},Z^{2},\cdots ,Z^{T}\}$$

where $Z_{i}^{t}$, $Z^{t}$ and *Z* are the dynamic representations of the *i*-th joint node of frame *t*, the *t*-th frame and the new sequence, respectively.

The embedding block consists of two convolution layers and two activation layers, as shown in Figure 3. These blocks extract features from 2D/3D position and motion tensors. The size of the convolution kernel is explained in Section 5.2.

### 4.2. Spatial Relative Transformer (SRT)

#### 4.2.1. Architecture

Different from the standard transformer, we prepend a virtual node to the graph of skeleton as the input. The virtual node gathers the global information from each joint node and scatters the information to all joint nodes; we named it the spatial-relay node. For the joint node and sptial-relay node, there are two corresponding connections: the inherent connections and the virtual connections.

##### Spatial Inherent Connections

**Figure 4:**
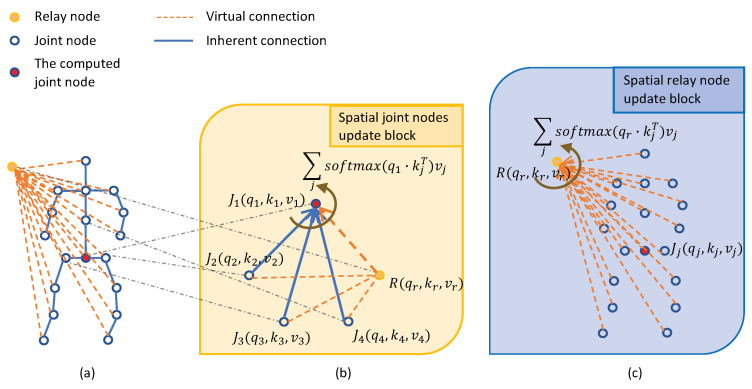
Illustration of the update blocks in a spatial relative transformer (SRT). The graph structure in SRT is described in (**a**). Updating operates on each joint node by obtaining local information from adjacent joint nodes and non-local information from the spatial-relay node in (**b**). Spatial-relay nodes are updated by scoring the contribution of each node, including spatial joint nodes and the spatial-relay node in (**c**).

As shown in Figure 4a, we establish inherent connections for all adjacent joints that have bone connections to preserve the inherent graph topology in skeletons. Such connections with prior knowledge allow each joint node to gather the local information from its adjacent joint nodes. Meanwhile, they enable joints to obtain more direct information from neighbors than non-adjacent joints, consistent with the general perception: neighbor joints are generally more important. A skeleton graph with *n* joint nodes has n−1 inherent connections.

##### Spatial Virtual Connections

The connections between every joint node and spatial-relay node are named after virtual connection (see in Figure 4a). Through the virtual connections, the sptial-relay node captures the global composition relationship; therefore, each joint node can obtain the information between the non-adjacent joint nodes. A skeleton graph with *n* joint nodes has *n* virtual connections. The combination of inherent and virtual connections makes the relative transformer obtain both local and global information. Compared with the standard transformer, the number of connections includes inherent and virtual connections. As a consequence, the model establishes a long-range dependency with low computational efficienvy and memory overhead.

#### 4.2.2. Implementation of SRT

In the spatial relative transformer model, each frame has its own relative transformer and we look at the model within one single frame. The model input $J_{graph}=\left\{J_{1}^{t},J_{2}^{t},\cdots ,J_{N}^{t}\right\}$ is a sequence of joint nodes at time *t*, where *N* is the number of nodes in this frame. $B_{J_{i}^{t}}$ is a set which contains the label of the adjacent joint nodes of $J_{i}^{t}$. Each node $J_{i}^{t}\left(R^{t}\right)$ has a query vector $q_{i}^{t}\left(q_{r}^{t}\right)$, a key vector $k_{i}^{t}\left(k_{r}^{t}\right)$ and a value vector $v_{i}^{t}\left(v_{r}^{t}\right)$.

##### Spatial Joint Nodes Update Block (SJU)

For each joint node, we calculate the strength of the correlations between them and their adjacent joint nodes (including the neighbor nodes $J_{B_{J_{i}^{t}}}^{t}$, the relay node $R^{t}$ and itself $J_{i}^{t}$) by taking the key dot product with the query vector, as shown in equation:

(11) $$\alpha_{ij}^{t}=q_{i}^{t}\cdot {k_{j}^{t}}^{T},i\in N,j\in \left[i;B_{J_{i}^{t}};r\right]$$

where $\alpha_{ij}^{t}$ represents the importance of node *j* on node *i*. Each neighbor node value vector $v_{j}^{t}$ is multiplied by the corresponding score $\alpha_{ij}^{t}$ and added up to update the joint node, as shown below:

(12) $$J_{i}^{t}=\sum_{j}softmax_{j}\left(\frac{\alpha_{ij}^{t}}{\sqrt{d_{k}}}\right)v_{j}^{t},\;i\in N,\;j\in \left[i;B_{J_{i}^{t}};r\right]$$

where $J_{i}^{t}$ is the updated result, aggregating local and global information. $d_{k}$ is the channel dimension of the key value (shown in Figure 4b).

In the model implement, the computations are implemented in matrix form. First, the $q_{i}^{t}$, $k_{j}^{t}$ and $v_{j}^{t}$ vectors are packed into $Q^{t}$, $K^{t}$ and $V^{t}$. Matrix $Q^{t}\in R^{C\times 1\times N}$ contains all joints’ query vectors for a single skeleton. Both matrix $K^{t}\in R^{C\times A\times N}$ and $V^{t}\in R^{C\times A\times N}$ contain all key vectors and value vectors, which correspond with an adjacent node matrix $M_{a}$ (the adjacent node matrix will be introduced in Section 4.4). *C* denotes the feature dimension; *N* is the number of joints in one single skeleton; *A* represents the maximum number of adjacent nodes. The attention in matrix form is defined as follows:

(13) $$Att\left(Q^{t},K^{t},V^{t}\right)=\sum_{i\in A}(softmax\left(\frac{mask(Q^{t}\circ K^{t})}{\sqrt{dk}}\right)\circ V^{t})$$

where ∘ is a Hadamard product and the mask operation removes the zeros taken by the padding operation.

##### Spatial Relay Node Update Block (SRU)

To ensure that the spatial-relay node better aggregates the information of all joint nodes, we also apply a transformer (see in Figure 4c). The importance of each joint node $\alpha_{rj}^{t}$ is computed by the query vector $q_{r}^{t}$ and the key vector $k_{j}^{t}$, as shown in the following:

(14) $$\alpha_{rj}^{t}=q_{r}^{t}\cdot {k_{j}^{t}}^{T},\;j\in \left[r;N\right]$$

The relay node $R^{t}$ is updated by:

(15) $$R^{t}=\sum_{j}softmax_{j}\left(\frac{\alpha_{rj}^{t}}{\sqrt{d_{k}}}\right)v_{j}^{t},j\in \left[r;N\right]$$

For the matrix key, all key vectors $k_{j}^{t}$ and value vectors $v_{j}^{t}$ are packed into matrix $K^{t}\in R^{C\times L}$ and $V^{t}\in R^{C\times L}$, respectively. The attention in matrix form is defined as follows:

(16) $$Att\left(q_{r}^{t},K^{t},V^{t}\right)=softmax\left(\frac{q_{r}^{t}\cdot K^{t}}{\sqrt{dk}}\right)\cdot {\left(V^{t}\right)}^{T}$$

where $q_{r}^{t}\in R^{1\times C}$ is the spatial relay node, · denotes the matrix product.

By alternately updating the joint nodes and the relay node, the spatial relative transformer will capture all the local and non-local information for an input graph. The overall update algorithm of the SRT is shown in the Algorithm 1.

### 4.3. Temporal Relative Transformer (TRT)

After designing a spatial relative transformer for each skeleton frame, we then formulate a temporal relative transformer to the skeleton sequence. Similar to the spatial relative transformer, the temporal relative transformer also consists of inherent connections and virtual connections by introducing a temporal-relay node.

Temporal Inherent Connections

**Figure 5:**
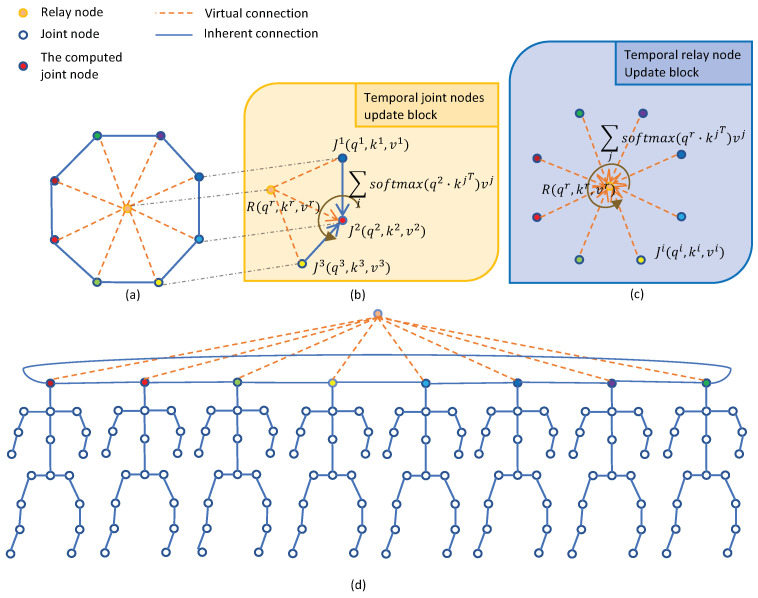
Illustration of the update blocks in temporal relative transformer (TRT). The same joint nodes in all sampled skeleton are connected in order and the joint nodes in the first and last frame are also connected in (**d**). This approach constitutes a ring-shaped structure, as shown in (**a**). Furthermore, each joint node and the temporal-relay node are updated by TJU in (**b**) and TRU in (**c**), respectively, similar to the methods in SRT.

Along the temporal dimension, the same joints across consecutive frames are treated as an input sequence into the model. Aside from the same joint nodes used between the adjacent, the joint nodes in the first and last frame are also connected, constituting a ring-shaped structure, as depicted in Figure 5d. A sequence formed by *n* nodes contains *n* inherent connections.

**Algorithm 1:** The update of spatial relative transformer

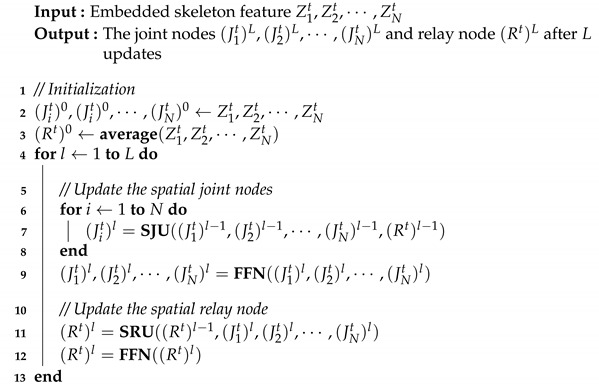



Temporal Virtual Connections

In the temporal relative transformer, each virtual connection links a joint node to the temporal-relay node, similar to the operation in spatial relative transformer. Hence, a sequence which contains *n* nodes has *n* virtual connections, as shown in Figure 5a. In a word, temporal relative transformer can capture the relationship between adjacent frames by the inherent connections, and the long-range relationship is captured by the virtual connections. This means that the semantic compositions are divided between the inherent connections and virtual connections, which enables the model to work without heavy pre-training. Furthermore, it reduces the number of connections from $n^{2}$ to 2n, where n is the skeleton sequence length.

#### 4.3.1. Implementation of TRT

In the temporal relative transformer model, each node is considered independently. As a result, the model is applied to the sequence $J=\{J_{v}^{1},J_{v}^{2},\cdots ,J_{v}^{T}\}$, which represents the same joint node from all frames in the sampled sequence. Each node $J_{v}^{i}\left(R_{v}\right)$ consists of a query vector $q_{v}^{i}\left(q_{v}^{r}\right)$, a key vector $k_{v}^{i}\left(k_{v}^{r}\right)$ and a value vector $v_{v}^{i}\left(v_{v}^{r}\right)$.

##### Temporal Joint Nodes Update Block (TJU)

As shown in Figure 5b, the joint node $J_{v}^{i}$ is updated by the relay node $r_{v}$, the same joint node in neighbor frames $\left(J_{v}^{i-1},J_{v}^{i+1}\right)$ and itself. Their score $\alpha_{v}^{ij}$ is expressed in the following:

(17) $$\alpha_{v}^{ij}=q_{v}^{i}\cdot {k_{v}^{j}}^{T},\;i\in T,j\in \left[i-1;i;i+1;r\right]$$

where $\alpha_{v}^{ij}$ represents the importance of the node in *j*-th frame on the same node in *i*-th frame.The joint nodes $J_{v}^{i}$ are updated by:

(18) $$J_{v}^{i}=\sum_{j}softmax_{j}\left(\frac{\alpha_{v}^{ij}}{\sqrt{d_{k}}}\right)v_{v}^{j},\;i\in T,j\in \left[i-1;i;i+1;r\right]$$

For the matrix key, all query vectors $q_{v}^{i}$ are packed into matrix $Q_{v}\in R^{C\times 1\times T}$, all key vectors $k_{v}^{j}$ are packed into matrix $K_{v}\in R^{C\times B\times T}$ and all key vectors $v_{v}^{j}$ are packed into matrix $V_{v}\in R^{C\times B\times T}$. *B* is the total number of *j*. The attention in matrix form is defined as follows:

(19) $$Att\left(Q_{v},K_{v},V_{v}\right)=\sum_{i\in B}(softmax\left(\frac{Q_{v}\circ K_{v}}{\sqrt{dk}}\right)\circ V_{v})$$

where ∘ denotes Hadamard product.

##### Temporal Relay Node Update Block (TRU)

The information of all the frames is aggregated to the temporal-relay node in Figure 5c by scaled dot-product attention, as expressed in the following:

(20) $$\alpha_{v}^{rj}=q_{v}^{r}\cdot {k_{v}^{j}}^{T},\;j\in \left[r;T\right]$$

(21) $$R_{v}=\sum_{j}softmax_{j}\left(\frac{\alpha_{v}^{rj}}{\sqrt{d_{k}}}\right)v_{v}^{j},\;j\in \left[r;T\right]$$

where $\alpha_{v}^{rj}$ is the attention score and $\frac{1}{\sqrt{d_{k}}}$ is a scaling factor. $R_{v}$ denotes the updated relay node.

For the matrix key, all key vectors $k_{v}^{j}$ and value vectors $v_{v}^{j}$ are packed into matrix $K_{v}\in R^{C\times T}$ and $V_{v}\in R^{C\times T}$, respectively. The attention in matrix form is defined as follows:

(22) $$Att\left(q_{v}^{r},K_{v},V_{v}\right)=softmax\left(\frac{q_{v}^{r}\cdot K_{v}}{\sqrt{dk}}\right)\cdot {\left(V_{v}\right)}^{T}$$

where $q_{v}^{r}\in R^{1\times C}$ is the temporal relay node, · denotes matrix product.

By alternately updating the relay node and the same joint node on all frames, the temporal relative transformer finally captures all the relationships in an input frame sequence. The overall update algorithm of the TRT is shown in Algorithm 2.
**Algorithm 2:** The update of temporal relative transformer
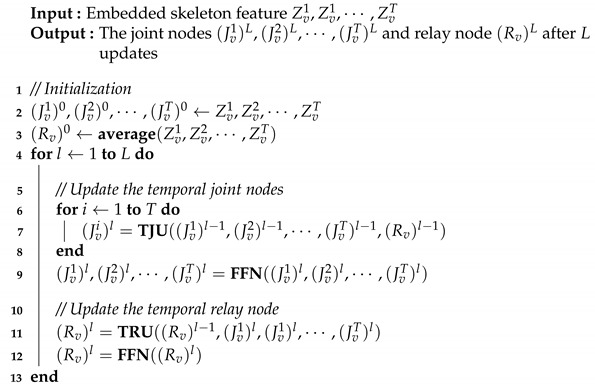


### 4.4. Detail of SRT and TRT

**Figure 6:**
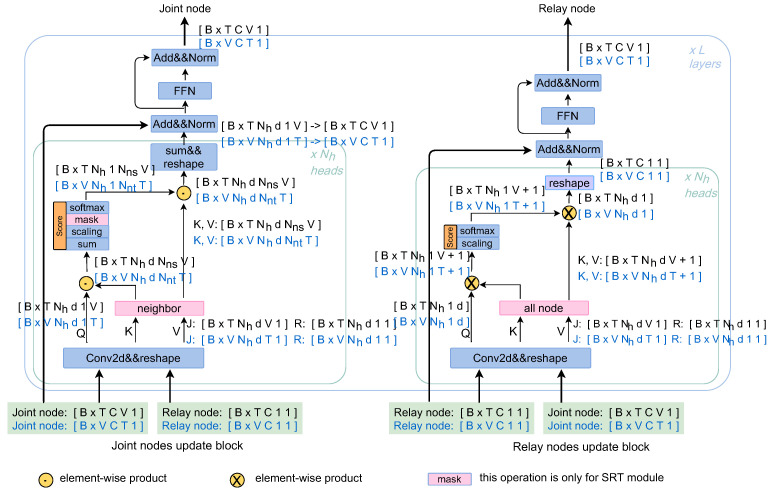
Illustration of the detail of the SRT module and TRT module. The tensor shapes with black font are for the SRT module and the blue are for the TRT module. It can be seen that the “mask” operation is only employed in SRT. In the SRT (TRT) module, the “neighbor” operation finds the adjacent nodes for each node according to Figure 4b and Figure 5b.

Given an input tensor of shape (B,C,V,T), where *B* is the number of batch sizes, *C* is the channel size of dynamic representation, *V* is the number of joint nodes in a skeleton and *T* is the number of frames in new sequence. As shown in Figure 6, we move the *T* dimension into the *B* dimension to get the new shape (B×T,C,V,1) in SRT module and move the *V* dimension into the *B* dimension to obtain the new shape (B×V,C,T,1) in the TRT module. The former implements the transformer on each frame independently, while the latter applies the transformer separately on each joint along the time dimension.

In a skeleton, each joint node generally has an unequal number of adjacent nodes. To solve this problem, we pad zeros to the nodes whose adjacent nodes are less than *A*. *A* denotes the maximum number of adjacent nodes. By doing this, we obtain an adjacent node matrix $M_{a}\in R^{N\times A}$, where *N* denotes the number of joint nodes in one skeleton. This operation is contained in step “neighbor” only in the SRT module, as shown in Figure 4b. Since these padding nodes are meaningless, we use a mask before the SoftMax operation, so that the attention mechanism avoids them.

Otherwise, multi-head attention with $N_{h}$ heads is applied to obtain richer information. The formula for attention translates into the following form:

(23) $$MultiHead\left(Q^{\prime },K^{\prime },V^{\prime }\right)=Concat\left(head_{1},\cdots ,head_{N}\right)$$

(24) $$head_{i}=softmax\left(\frac{\left(Q^{\prime }W_{i}^{Q}\right){\left(K^{\prime }W_{i}^{K}\right)}^{T}}{\sqrt{d_{k}^{i}}}\right)V^{\prime }W_{i}^{V},\;i\in \left[1,N_{h}\right]$$

(25) $$X=X^{\prime W_{i}^{X}},\;X\in \left\{Q,K,V\right\}$$

where $Q^{\prime },K^{\prime },V^{\prime }$ is the input matrixes, and $W_{i}^{Q}$, $W_{i}^{K}$, $W_{i}^{V}$ are learnable parameter matrices. $d_{k}^{i}$ is the channel dimension of *K* for $head_{i}$.

## 5. Experience

In this section, we evaluate the performance of the proposed MSST-RT on three large-scale skeleton datasets, namely, NTU RGB+D [29], NTU RGB+D 120 [30] and UAV-Human [31]. Extensive ablation studies are conducted to validate the contributions of different components in our model. Finally, we visualize the attention probabilities in SRU and TRU block to prove the effectiveness of the proposed ST-RT model.

### 5.1. Datasets

#### 5.1.1. NTU RGB+D

The data in NTU RGB+D are collected in the lab environment by three Microsoft Kinect v2 cameras, which are widely used for skeleton-based action recognition tasks. They contain 56,680 skeleton sequences performed by 40 subjects, covering a total of 60 action classes. Each sequence contains, at most, two subject skeletons and each skeleton is composed of 25 joints. As the authors of this dataset recommended, we used two evaluation protocols, namely cross-subject (CS) and cross-view (CV). In the CS setting, 20 subjects were used for training and the rest for testing. In the CV setting, the sequences captured by camera 2 and camera 3 were used for training while the rest were used for testing.

#### 5.1.2. NTU RGB+D 120

NTU RGB+D 120 is an extension of NTU RGB+D, whose action classes are increased to 120, subjects are increased to 106 and sequences are increased to 114,480. There are also two benchmarks, namely, cross-subject (C-subject) and cross-setup (C-setup). In the C-subject setting, 53 subjects are used for training and the rest for testing. In the C-setup setting, the dataset is divided by the parity of the setup IDs into two group, with one used for training and another used for testing.

#### 5.1.3. UAV-Human

UAV-Human is a new dataset which is of significance fo practical UAV application scenarios. This dataset covers different types of human behavior and is collected by a flying UAV in multiple urban and rural districts in the day and night. It contains 155 activity classes in six different modalities including RGB, depth, IR, fisheye, night-vision, and skeleton sequences. There are 22,476 frames with the 2D positions of 17 major key-points of the human body for skeleton-based recognition, 16,169 frames for training and 6307 frames for testing.

### 5.2. Implementation Details

All experiences are performed on the Pytorch platform. Similar to [20], we used the Adam optimizer with β=[0.9,0.98] and $\epsilon =10^{-9}$. Moreover, a two-phase training strategy was utilized to make the model convergence faster and more stable. The gradual warmup strategy linearly increased the learning rate from $4\times 10^{-7}$ to 0.0005 for the first training phase (the first 700 steps), while natural exponential decay with the weight decay of 0.9996 proportionally decreased the learning rate for the second phase. During training, the batch sizes for NTU60, UAV-Human and NTU120 were 64, 64 and 100, respectively, and the total training epochs was set to 30. Label smoothing of value $\epsilon_{ls}=0.1$ was employed for all experiences.

In terms of data processing, the original skeleton coordinates of each frame were replaced with its displacement relative to the first frame. The actions, which contained two skeletons, such as hugging, were divided into two sequences so that each sequence contained only one. Moreover we employed data argumentation by randomly rotating the 3D skeletons to create more variational samples, which could improve the generalization ability of the network.

Raw position, short motion, long motion and adaptive motion were embedded from dimension space 2 (for UAV-Human) or 3 (for NTU and NTU120) to a dimension space of (C) 64 by the first convolution in all the experiments. Through the second convolution, they were embedded from a space of dimension (C) 64 to a space of dimension $\left(C_{1},C_{2},C_{3},C_{4}\right)$ 256, 256, 128 and 128, respectively. Note that the weights of the convolution layers are not shared among them.

### 5.3. Comparison to State of the Art

The performance of the proposed MSST-RT is compared with other state-of-the-art methods on the NTU60, NTU120 and UAV-Human datasets in Table 1, Table 2 and Table 3, respectively. The contents of the “ST-RT()” brackets represent the number of sampled frames (10 or 20) and the type of input skeleton data (joint or bone), respectively.

As shown in Table 1, MSST-RT achieves a good performance, with 88.43% and 93.21%, respectively, for Cross Subject (CS) and Cross View (CV) settings of NTU RGB+D 60. It is worth noting that STA-LSTM and TS-SAN both adopt the attention mechanism, which is similar to the model idea that we proposed. The difference is that STA-LSTM uses LSTM in addition to the attention mechanism, while our proposed model only uses the attention mechanism. Moreover, our model outperforms this model by 14.96% with CS and 12.0% with CV. Cmparing our model with TS-SAN, the attention mechanisms in ST-RT are employed not only between video frames, but also between the joint nodes of the skeletons. Our model outperforms it for 1.23% with CS and 0.51% for CV.

As shown in Table 2, the proposed MSST-RT achieves the best performance, with 79.33% for the C-subject setting and 82.30% for the C-setup setting. Most of the methods we compare are reported in [22].

As shown in Table 3, the proposed MSST-RT achieves the best performance, with 41.12%, and outperforms the second place by 3.24%. UAV-Human is a new dataset, released in 2021, and we compare the performance of our model with the results reported in [31]. Single-stream ST-RT, namely “ST-RT(Seg = 20, joint)”, outperformed all methods.

### 5.4. Ablation Study

Ablation studies of performance were performed by the proposed ST-RT model on the NTU RGB+D dataset with the CS setting. Firstly, two normalization methods were chosen for comparison to provide faster training and better performance. Moreover, we compared dynamic representations with different motion combination methods and different networks with different stream combination methods. Finally, the models were investigated with different hyper-parameters, such as the number of sampled frames, layers and heads. Furthermore, an ablation study regarding computation cost was based on UVA-Human.

#### 5.4.1. The Effect of Different Normalization Methods

**Figure 7:**
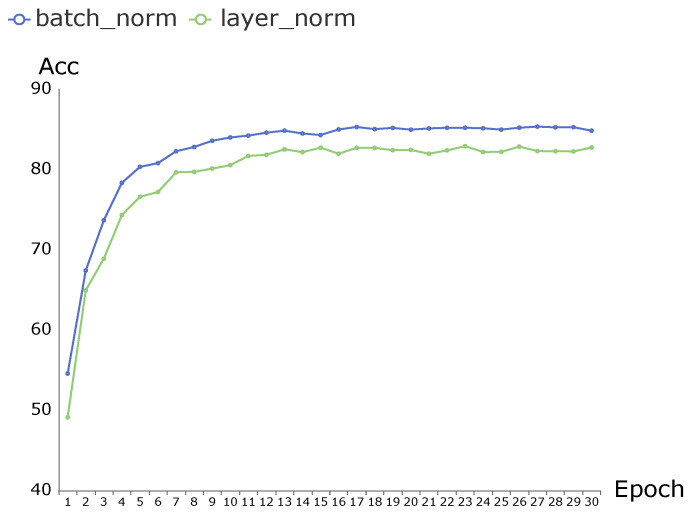
Difference in terms of accuracy and convergence speed between an ST-RT with batch normalization and an ST-RT with layer normalization.

Normalization is used in both dynamic representation modules and relative transformer modules. The normalization method in a relative transformer module can either use layer normalization or batch normalization, while the normalization method in the dynamic representation module only can use layer normalization. Table 4 shows that “ST-RT (BatchNorm)” is superior to “ST-RT (LayerNorm)” by 2.03%. The accuracy of the two normalization methods is shown in Figure 7 as a function of the epoch. The accuracy of “ST-RT (BatchNorm)” increases faster than that of “ST-RT (LayerNorm)” and it is stable at a higher value. The experiments demonstrate that batch normalization provides faster training times and a better performance.

#### 5.4.2. The Effect of Dynamics Representation with Different Combination

To demonstrate that each motion provides different information, we compare our ST-TR model with three different models whose DR module only contains two motions, and show the results in Table 5. “ST-RT(w/o X motion)” denotes that the “X” motion is removed from the DR module in the ST-RT model, “X” is “short”, “long” or “adaptive”. The accuracy of the ST-TR model has a 0.60% reduction, a 1.17% reduction and a 0.69% reduction when it is without short motion, long motion or adaptive motion, respectively. This means that the information captured from each motion can complement the other two motions. According to the reduction in accuracy, we conclude that long motion contains richer dynamic information compared to short motion.

Table 6 shows the effect of sampling frame numbers on model performance. From the results of “ST-RT(Seg = 15)” to “ST-RT(Seg = 10 && Seg = 15)”, we observe that the model underperforms in both superfluous and insufficient frame scenarios. The experiments demonstrate that the model achieves the best performance with 86.46% when sampling 20 frames. In addition, we combined networks with different sampling frames (shown in the last four rows of Table 6, i.e., “ST-RT(Seg = 10 && Seg = 15)” denotes the combination of the network with 10 sample frames and the network with 15 sample frames). “ST-RT(Seg = 10 && Seg = 20)” outperforms other combinations with 87.48%, and this optimal parameter was applied in MSST-RT.

#### 5.4.3. The Effect of Number of Layers and Multi-Heads in ST-RT Model

The results of ST-RT model with a different number of layers and heads are shown in Table 7. “LX” represents the SRT (TRT) module with X layers; each layer contains one SJU (TJU) block, one SRU (TRU) block and two FFNs. “HY” represents each attention mechanism in ST-RT model with Y multi-heads. By comparing the number of heads, we observe that more multi-heads always results in better performance, especially when the model’s heads increase from 4 to 8. However, the affect of head number peaks when it increases to 12, and excessive heads can make the accuracy of the lightweight model decrease (shown in “ST-RT (L2H8)” and “ST-RT (L2H12)”). From the results of “ ST-RT (L2H8)”, “ ST-RT (L3H8)” and “ ST-RT (L4H8)”, we conclude that the model underperforms if the number of layers is too high or too low. In sum, number of layers and heads is set as 3 and 8, respectively, for the proposed model, considering the number of parameters and the accuracy.

#### 5.4.4. The Effect of Dynamics Representation with Different Combination

The speed and accuracy of different variants of MSST-RT with different numbers of streams are shown in Table 8. When using the same number of frames, the joint stream has a similar computation cost to the bone stream. The computation cost increases with the increase in the number of frame. In the variants with two streams, "ST-RT(s3+s4)" achieves the best performance, which is slightly lower than MSST-RT, and the computation cost is half of MSST-RT’s. Hence, the stream numbers can be reduced if there are concerns regarding computation cost.

### 5.5. Visualization of SRU and TRU

Our model applies the attention mechanism when updating joint nodes and virtual nodes in both the spatial and temporal dimensions. The attention response is visualized from the last SRU block in the Spatial Relative Transformer (SRT) and the last TRU block in the Temporal Relative Transformer (TRT).

**Figure 8:**
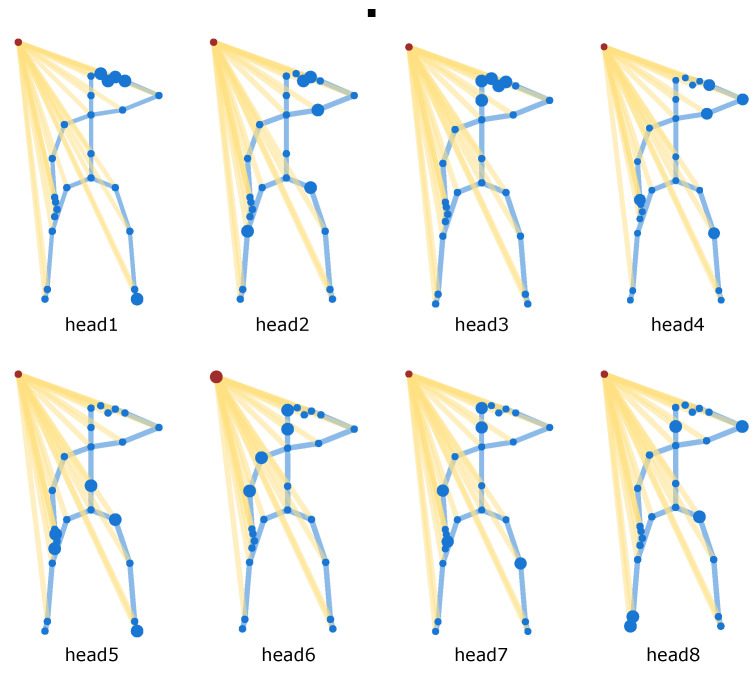
Visualization of the attention responses of the last SRU block in ST-RT model the action is salute.

**Figure A1:**
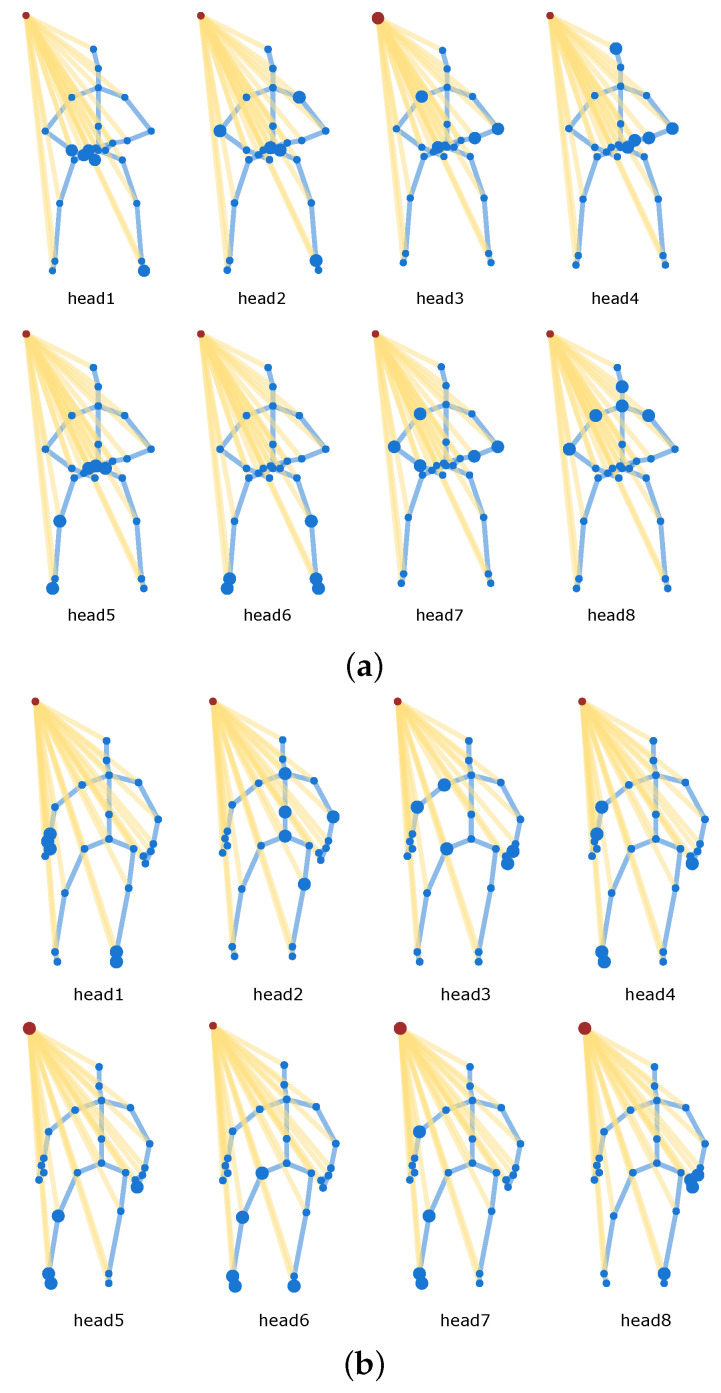
Visualization of the attention responses of the last SRU block in ST-RT model with respect to two actions, i.e., clapping (**a**) and kicking something (**b**).

The action salute is selected to visualize the attention response for eight multi-heads from the last SRU layer in Figure 8. The red circles represent the spatial-relay nodes and the blue circles represent the joint nodes. The blue lines represent the inherent connections and the yellow lines represent the virtual connections. We zoom in on the five nodes with the highest corresponding attention values, and the other nodes are shown by small circles. The attention response of each head is different and head1, head2, head3 and head4 all focus mainly on the left hand. This suggests that the attention mechanism works in a similar way to human perception. Otherwise, the actions clapping and kicking something are visualized in Appendix A, which shows that different actions focus on different nodes. Both left and right hands are important for “clapping”, so most heads focus on hands in Figure A1a. For “kicking something” in Figure A1b, only the right foot is of great value.

**Figure 9:**
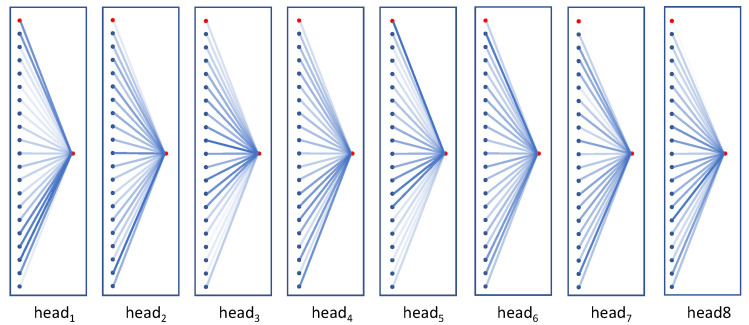
Visualization of the attention responses in the last TRU block in the ST-RT model.

Figure 9 shows the attention response for the action “salute” for eight multi-heads from the last TRU layer. The red circles represent the temporal-relay nodes (the left one in the sequence is the node before updating and the right one is the node after updating) and the 20 blue circles represent the eighth joint nodes from 20 sampled frames. The transparency of lines indicates the intensity of the attention response. The brighter color denotes the higher response. This shows that different frames are noted in different heads, and the information from each frame, including the temporal-relay node itself before being updated, is converged to the temporal-relay node. We can see that the temporal-relay node in last layer (the left red node in the sequence) receives a large amount of attention from head1 and head5.

## 6. Conclusions

In this work, transformer architecture is introduced to establish a long-range dependence instead of graph convolution. Significantly, MSST-RT relies on transformer architecture instead of recurrence, LSTM or graph convolution, which is a full attention model. Specifically, we propose a novel architecture based on standard transformer and named a relative transformer. This compensates for the deficiencies of the standard transformer while retaining the inherent topology of the skeleton, and significantly reduces computational complexity. The architecture, meanwhile, makes it possible for this to work without heavy pre-training. Furthermore, the relative transformer module evolves into a spatial relative transformer and temporal relative transformer, respectively, to extract spatial-temporal features. In addition, the DR module combines multi-scale motion information to adaptively recognize actions with different durations and different ranges of motion. Finally four streams with an ST-RT module, with four dynamic data streams, are fused to complement each other, realizing the further enhancement of performance. The final network, MSST-RT, achieves a state-of-the-art performance in skeleton-based action recognition on NTU RGB+D, NTU RGB+D 120 and UAV-Human. It is worth noting that single-stream ST-RT outperformed other methods on UAV-Human. It outperformed TS-SAN [37], which also adopted attention architecture. The results of attention response visualization verify the effectiveness of the proposed model for skeleton-based action recognition tasks. 

## Figures and Tables

**Table 1:** Performance comparison on NTU RGB+D.

Methods	Year	CS(%)	CV(%)
HBRNN-L [9]	2015	59.1	64.0
Part-Aware LSTM [29]	2016	62.9	70.3
ST-LSTM+Trust Gate [32]	2016	69.2	77.7
Two-stream RNN [7]	2017	71.3	79.5
STA-LSTM [33]	2017	73.4	81.2
VA-LSTM [34]	2017	79.4	87.6
ST-GCN [17]	2018	81.5	88.3
DPRL+GCNN [19]	2018	83.5	89.8
HCN [35]	2018	86.5	91.9
AS-GCN [36]	2019	86.8	94.2
TS-SAN [37]	2020	87.2	92.7
ST-RT(seg = 10, joint)	-	86.20	91.03
ST-RT(seg = 10, bone)	-	85.10	90.51
ST-RT(seg = 20, joint)	-	86.44	92.46
ST-RT(seg = 20, bone)	-	85.61	90.61
MSST-RT	-	88.43	93.21

**Table 2:** Performance comparison for NTU RGB+D 120.

Methods	Year	CS(%)	CV(%)
Part-Aware LSTM [29]	2016	25.5	26.3
ST-LSTM + Trust Gate [32]	2016	55.7	57.9
GCA-LSTM [8]	2017	58.3	59.2
Two-Stream GCA-LSTM [38]	2017	61.2	63.3
RotClips+MTCNN [39]	2018	64.6	66.9
SGN [40]	2020	79.2	81.5
ST-RT(seg = 10, joint)	-	76.22	80.26
ST-RT(seg = 10, bone)	-	75.09	78.37
ST-RT(seg = 20, joint)	-	76.67	79.58
ST-RT(seg = 20, bone)	-	76.45	76.85
MSST-RT	-	79.33	82.30

**Table 3:** Performance comparison on UAV-Human.

Methods	Year	Accuracy (%)
ST-GCN [17]	2018	30.25
DGNN [18]	2019	29.90
2s-AGCN [28]	2019	34.84
HARD-Net [41]	2020	36.97
Shift-GCN [42]	2020	37.98
ST-RT(seg = 10, joint)	-	36.83
ST-RT(seg = 10, bone)	-	35.86
ST-RT(seg = 20, joint)	-	38.73
ST-RT(seg = 20, bone)	-	37.18
MSST-RT	-	41.22

**Table 4:** Accuracy comparison of the ST-RT models between two normalization methods in ST-RT model.

Norm Methods	CS(%)
ST-RT (LayerNorm)	84.42
ST-RT (BatchNorm)	86.46

**Table 5:** Accuracy comparison of the ST-RT models with different motion and their combination. “w/o” equals without.

Methods	CS (%)
ST-RT(w/o short motion)	85.86
ST-RT(w/o long motion)	85.29
ST-RT(w/o adaptive motion)	85.77
ST-RT	86.46

**Table 6:** Accuracy comparison of the ST-RT models with different sampling strategies and their combination. “X && Y” means the fusion of the model with X sampling strategy and the model with Y sampling strategy.

Methods	CS (%)
ST-RT (Seg = 10)	86.20
ST-RT (Seg = 15)	86.07
ST-RT (Seg = 20)	86.46
ST-RT (Seg=25)	85.21
ST-RT (Seg = 10 && Seg = 15)	86.93
ST-RT (Seg = 10 && Seg = 20)	87.74
ST-RT (Seg = 10 && Seg=25)	87.00
ST-RT (Seg = 15 && Seg = 20)	87.40
ST-RT (Seg = 15 && Seg = 25)	86.92
ST-RT (Seg = 20 && Seg = 25)	87.41

**Table 7:** Accuracy comparison for the ST-RT models with different sampling strategies, and their combination. “X && Y” means the fusion of the model with X sampling strategy and the model with Y sampling strategy.

Methods	CS (%)
ST-RT (L2H4)	85.60
ST-RT (L2H8)	86.26
ST-RT (L2H12)	86.15
ST-RT (L3H4)	84.76
ST-RT (L3H8)	86.46
ST-RT (L3H12)	86.81
ST-RT (L4H4)	81.71
ST-RT (L4H8)	85.21
ST-RT (L4H12)	85.35

**Table 8:** The effect of number of streams in the ST-RT Model on UAV-Human. “s1” is the stream with 10 frames, joint frames, “s2” is the stream with 10 frames, bone frames, “s1” is the stream with 20 frames, joint frames, “s1” is the stream with 20 frames, bone frames. Furthermore, “ST-RT(X + Y)” is the fusion of the model with X stream and the model with Y stream. “Computation cost” means average second per epoch on the train set.

Methods	Accuracy (%)	Computation Cost(s)
ST-RT(s1)	36.83	305.00
ST-RT(s2)	35.86	312.28
ST-RT(s3)	38.73	447.51
ST-RT(s4)	37.18	450.94
ST-RT(s1 + s2)	39.05	617.28
ST-RT(s3 + s4)	41.18	898.45
ST-RT(s1 + s3)	39.46	752.51
ST-RT(s2 + s4)	38.20	763.22
MSST-RT	41.22	1515.73

## Data Availability

NTU RGB+D and NTU RGB+D 120 action recognition dataset: https://github.com/shahroudy/NTURGB-D, accessed on 30 June 2021. UAV-Human dataset: https://github.com/SUTDCV/UAV-Human, accessed on 30 June 2021.

## Abbreviations

The following abbreviations are used in this manuscript:

MSST-RT	Multi-stream spatial-temporal relative transformer
ST-RT	spatial-temporal relative transformer
FFN	feed forward neural network
DR	dynamic representation
SRT	spatial relative transformer
SJU	spatial joint nodes update block
SRU	spatial relay node update block
TRT	temporal relative transformer
TJU	temporal joint nodes update block
TRU	temporal relay node update block
